# Global knowledge structure and thematic evolution in inflammation-associated gastric cancer epigenetics: a multidatabase science-mapping study with nested latent Dirichlet allocation and independent NMF validation

**DOI:** 10.3389/fimmu.2026.1946189

**Published:** 2026-09-25

**Authors:** Zhiwen Li, Xiaofeng Wang, Rui Li, Yisen Hou, Wenhao Chen

**Affiliations:** Department of Oncology Surgery, Xi’an No. 3 Hospital, The Affiliated Hospital of Northwest University, Xi’an, Shaanxi, China

**Keywords:** Citespace, DNA methylation, epigenetics, gastric cancer, inflammation, latent Dirichlet allocation, multi-database bibliometrics, non-coding RNA

## Abstract

**Background:**

Inflammation-associated epigenetic regulation links microbial exposure and chronic mucosal injury to gastric carcinogenesis, immune remodeling, molecular heterogeneity, and treatment resistance. Previous science-mapping studies have commonly relied on a single bibliographic database, which may introduce platform-specific coverage bias.

**Methods:**

Web of Science Core Collection (WoSCC), PubMed, and Scopus were searched for English-language articles and reviews published from 2000 through July 2026 using harmonized concepts for gastric malignancy, inflammation, and epigenetic regulation. Database-specific screening yielded 1, 407 WoSCC, 1, 073 PubMed, and 1, 935 Scopus records. Records were harmonized and deduplicated by DOI, PMID, normalized title, publication year, and author information. The resulting multi-database corpus was analyzed with CiteSpace, VOSviewer, and R. A nested latent Dirichlet allocation (LDA) analysis was retained on the internally consistent WoSCC subset: 1, 406 records underwent relevance screening and 808 entered the final topic model. As an independent robustness analysis, non-negative matrix factorization (NMF) was applied to title-and-abstract text from all 2, 933 unique publications.

**Results:**

The three searches contributed 4, 415 records and 2, 933 unique publications after removal of 1, 482 duplicate occurrences. The corpus covered 885 sources, showed an annual growth rate of 14.7%, and peaked at 346 publications in 2021. Standardized country data identified China as the largest contributor (1, 549 documents), whereas the United States had the strongest international connectivity (total link strength [TLS], 405). Nanjing Medical University was the leading institution by output (70 documents), and Toshikazu Ushijima was the most prolific author (25 documents). Multi-database keyword mapping identified gastric cancer, microRNA, cell proliferation, signal transduction, apoptosis, inflammation, DNA methylation, tumor microenvironment, STAT3, and long non-coding RNA as central concepts. Burst detection showed an early emphasis on DNA methylation, Helicobacter pylori, and RNA interference, followed by recent cross-cancer and functional terms. The WoSCC-based six-topic LDA model remained balanced in prevalence (15.93%-18.26%) and showed significant declines in H. pylori-inflammation-precancer and DNA hypermethylation-silencing topics, with concurrent growth in immune-lncRNA, microbiota/virus-associated ncRNA, and exosomal ncRNA-cytokine themes. Independent NMF analysis of the full multi-database corpus recovered broadly concordant domains involving H. pylori-associated inflammation, ncRNA-related signaling, immune and exosomal microenvironmental processes, and DNA methylation-related epigenetic regulation.

**Conclusions:**

Multi-database integration substantially expanded the observable literature and indicated a broad shift in research attention from infection-associated methylation toward ncRNA-centered, immune, microenvironmental, and resistance-related regulatory systems. National collaboration was broader than institutional and author-level integration. The nested LDA analysis quantified the long-term reallocation of thematic attention, while independent NMF validation across the full multi-database corpus supported the robustness of the principal thematic domains.

## 1 Introduction

Gastric cancer is a biologically heterogeneous disease shaped by microbial exposure, chronic inflammation, host susceptibility, somatic alterations, and epigenetic reprogramming (1–3). Epigenetic mechanisms translate environmental and inflammatory cues into persistent changes in transcription without altering the underlying DNA sequence. DNA methylation, histone modification, chromatin remodeling, and non-coding RNA networks influence malignant transformation, lineage plasticity, immune evasion, invasion, metastasis, and treatment response (3–8).

The field has evolved markedly over the past quarter century. Early investigations concentrated on CpG-island hypermethylation, promoter silencing, and the epigenetic field defect induced by chronic Helicobacter pylori infection. Subsequent work expanded toward Epstein-Barr virus-associated methylation, molecular classification, microRNAs, long non-coding RNAs (lncRNAs), circular RNAs, competing endogenous RNA networks, extracellular vesicles, tumor immunity, and resistance to systemic therapy (9–13). The increasing volume and mechanistic diversity of this literature make conventional narrative synthesis alone insufficient for reconstructing the field’s global architecture and temporal development (14).

Science mapping can characterize publication growth, collaboration networks, keyword architecture, and emerging research fronts, whereas probabilistic topic modeling can infer latent themes from titles, abstracts, and keywords (15–21). However, database coverage is not interchangeable. WoSCC, Scopus, and PubMed differ in journal inclusion, biomedical indexing, author-address structure, and citation metadata (22, 23). A single-database design may therefore overrepresent platform-specific journals or terminology, whereas multi-database integration increases coverage but requires transparent deduplication and field harmonization (24, 25).

This study was designed as an original research-on-research investigation with three complementary analytical layers. First, we integrated WoSCC, PubMed, and Scopus to examine publication growth, database overlap, collaboration networks, keyword structure, and burst dynamics. Second, we retained the previously validated WoSCC text corpus for a nested LDA analysis, thereby preserving an internally consistent vocabulary space while quantifying latent-topic prevalence, temporal trends, assignment uncertainty, and compositional relationships. Third, we applied NMF to the title-and-abstract text of the full 2, 933-publication multi-database corpus as an independent robustness analysis to assess whether the principal LDA-derived thematic domains remained visible under broader database coverage and a mathematically distinct topic-modeling framework. We aimed to reconstruct the global knowledge structure and thematic evolution of inflammation-associated gastric cancer epigenetics, evaluate the robustness of the major latent themes across analytical layers, and identify current structural and translational priorities.

## 2 Materials and methods

### 2.1 Study design and multi-database search

This retrospective research-on-research study analyzed bibliographic metadata from WoSCC, PubMed, and Scopus. The searches were conducted and updated between July 14 and July 21, 2026, and covered publications indexed from January 1, 2000, through the respective search dates. The three database strategies used harmonized concept blocks for (i) gastric malignancy, including gastric cancer, gastric carcinoma, gastric adenocarcinoma, stomach cancer, gastric neoplasm, and gastric carcinogenesis; (ii) inflammation, including inflammatory terms, cytokines, IL-6, TNF-alpha, IL-1beta, NF-kappa B, and JAK/STAT3; and (iii) epigenetic regulation, including DNA methylation, promoter methylation, histone modification, chromatin regulation, epigenomics, microRNA, lncRNA, circRNA, and other non-coding RNA classes. Database-specific syntax is summarized in **Supplementary Table S1**.

The three concept blocks were intentionally combined with the AND operator because the prespecified scope of this study was inflammation-associated epigenetic research in gastric cancer rather than the entire gastric cancer epigenetics literature. This intersection-based strategy was designed to increase conceptual specificity for studies linking inflammatory or microbial processes with epigenetic regulation in gastric malignancy. We recognize that this approach favors specificity over sensitivity and may exclude relevant gastric cancer epigenetic studies in which inflammatory mechanisms were not explicitly represented in the indexed title, abstract, or keyword fields; accordingly, the findings should be interpreted within this predefined inflammation-associated scope.

Eligibility was restricted to English-language journal articles and reviews published from 2000 through 2026. Retractions and retraction notices were excluded. Nonexclusive status labels, such as Early Access, did not lead to exclusion when the record was otherwise an article or review. The final database-specific exports comprised 1, 407 WoSCC, 1, 073 PubMed, and 1, 935 Scopus records. WoSCC records included full bibliographic fields and cited references, PubMed records were exported in tagged PubMed format, and Scopus records were exported as CSV with bibliographic information, abstracts, indexed keywords, affiliations, and references where available.

### 2.2 Cross-database harmonization and deduplication

The three exports were parsed into a unified record structure containing title, year, authors, first author, source title, DOI, PMID, WoS accession number, Scopus EID, abstract, author keywords, database-indexed keywords, MeSH terms, affiliations, countries, document type, and database provenance. DOI strings were normalized by removing resolver prefixes and punctuation and converting to lowercase. Deduplication was conducted hierarchically using exact DOI matching, exact PMID matching, standardized title with publication year, and high-similarity title matching supported by first author and source information. Ambiguous near-matches with conflicting persistent identifiers were retained as separate records. This approach followed established principles of transparent multi-database deduplication (25).

For duplicate groups, complementary metadata were merged rather than discarding nonidentical fields. WoSCC contributed accession numbers and Keywords Plus, PubMed contributed PMID and MeSH terms, and Scopus contributed EID and indexed keywords. Each merged record retained a database-origin label. WoSCC and Scopus citation counts were preserved in separate fields and were not summed because platform-specific citation coverage is not directly comparable. The primary multi-database corpus contained one row per unique publication.

### 2.3 Descriptive indicators and publication-growth analysis

The merged corpus was summarized using publication year, source title, authorship, document type, database origin, and keyword fields. Annual scientific production was calculated from observed publication counts. The annual growth rate and Bradford source zones were obtained from bibliometric summary analysis in R. Exploratory logistic cumulative-growth and annual life-cycle models were fitted to characterize growth patterns and a potential transition toward field maturation; these curves were interpreted descriptively rather than as definitive forecasts. Because the 2026 search period was incomplete, the 2026 count was treated as a partial-year observation.

### 2.4 Collaboration-network analysis

CiteSpace 6.4.R2 was used to generate multi-database collaboration networks at the country/region, institution, and author levels. The analysis covered 2000–2026 with one year per time slice. Nodes were selected using the g-index with k=6; additional parameters were LRF = 3.0, L/N=10, LBY = 5, and e=1.0. Minimum spanning tree pruning was applied to reduce visual redundancy while retaining the main backbone. Node size represented publication activity, links indicated co-authorship, concentric colors encoded time, and purple rims indicated higher betweenness centrality. Network density and the largest connected component were used to assess integration.

Author names, country aliases, and organization names were harmonized for the tabulated rankings. Author-name harmonization was limited to normalization of bibliographic name strings and did not constitute definitive person-level disambiguation. ORCID identifiers and Scopus Author IDs were not consistently available across the integrated records and were therefore not used for systematic person-level disambiguation; comprehensive manual identity verification was not performed. Therefore, records sharing common surnames and initials could not always be reliably distinguished, and author-level rankings were interpreted conservatively, particularly for common names. Country labels such as China/People’s R China and United States/USA were consolidated. Generic department-only or incomplete organization fragments were excluded from the institution ranking. Total link strength (TLS) was used as a measure of aggregate collaboration connectivity and was not interpreted as citation impact. Because citation coverage differed across databases, the multi-database collaboration tables do not report pooled citations.

### 2.5 Keyword co-occurrence, temporal overlay, density mapping, and burst detection

VOSviewer was used to construct co-occurrence, temporal overlay, and density maps from the harmonized multi-database term set (20). The source fields included WoSCC author keywords and Keywords Plus, Scopus author and indexed keywords, and PubMed other terms and MeSH headings. Full counting was applied. Common variants were normalized with a thesaurus, including gastric-cancer/gastric cancer, H. pylori/Helicobacter pylori, lncRNA/long non-coding RNA, and NF-kappa B/NF-kB. Terms with at least 50 occurrences were used for the principal map, while a lower-threshold network was retained for exploratory review.

CiteSpace burst detection was used to identify the 25 terms with the strongest increases in occurrence. Burst strength, onset, endpoint, and duration were recorded. Because the pooled term set includes database-generated indexing terms, generic demographic, document-type, and cross-cancer terms were interpreted as indexing or methodological signals rather than automatically as gastric-cancer-specific research fronts.

### 2.6 Nested WoSCC corpus for topic modeling and relevance screening

The LDA analysis was conducted on the WoSCC subset rather than the merged three-database corpus. This nested design preserved a consistent combination of title, abstract, author-keyword, and Keywords Plus fields and enabled direct continuity with the previously validated topic-modeling workflow. The LDA findings were therefore interpreted as a content-focused secondary analysis nested within the broader multi-database science-mapping study, not as a replacement for the multi-database descriptive results.

Among the 1, 407 WoSCC publications, one record could not be converted into an analyzable text document. The remaining 1, 406 records underwent relevance screening. Missing title, abstract, and keyword fields were replaced with empty strings. A record was retained when a gastric-cancer-related term appeared in the title or author keywords, or occurred at least twice in the abstract. A secondary rule retained studies of gastric precancerous lesions, gastric mucosal epigenetics, and H. pylori-associated epigenetic mechanisms when gastric cancer was a clear etiological or translational context. This procedure retained 808 records for LDA and excluded 598 records whose principal textual focus was not gastric cancer.

### 2.7 Text preprocessing and document-term matrix

Titles, abstracts, author keywords, and Keywords Plus were combined into one text field per record. Text was converted to lowercase; punctuation and noninformative characters were standardized; standard English stop words and common methodological terms were removed; and selected multiword biomedical expressions were retained with underscore notation. Sparse and very low-frequency terms were removed. The final document-term matrix contained 808 documents, 2, 315 terms, and 77, 004 nonzero entries.

### 2.8 LDA model development, selection, and interpretation

LDA models were estimated in R with Gibbs sampling implemented through the topic-modeling framework (21). Candidate solutions from K = 4 to K = 12 were compared using perplexity, UMass coherence, minimum dominant-topic size, balance across topic sizes, random-seed stability, and substantive interpretability. Perplexity was interpreted as lower-is-better, UMass coherence as higher-is-better, and minimum topic size as larger-is-better. Random-seed stability was assessed for K = 5, K = 6, and K = 7 across seeds 42, 7, and 2026 using matched-topic Jaccard similarity of high-probability terms (26).

K=6 was selected as the final compromise. At K = 6, perplexity was 861.73, UMass coherence was -1.544, and the minimum dominant-topic size was 93 documents (11.51%). Mean random-seed stability was 0.546 for K = 6, close to 0.554 for K = 5 and greater than 0.464 for K = 7. The final model used seed 7. Topic labels were assigned after joint review of high-probability terms, the 20 most representative documents per topic, and biomedical coherence.

### 2.9 Independent multi-database validation using NMF

As an independent robustness analysis, non-negative matrix factorization (NMF) was applied to the integrated corpus of 2, 933 unique publications. To minimize heterogeneity arising from database-specific indexing fields, only text fields shared across WoSCC, PubMed, and Scopus were used, comprising publication titles and abstracts. Abstract text was available for 2, 932 records; the single record without an available abstract was represented by its title alone. Text was lowercased and normalized, and selected biomedical expressions and variants were harmonized, including *H. pylori*, lncRNA, miRNA, ncRNA, NF-κB, DNA methylation, EBV, PD-1/PD-L1, and specific miRNA identifiers. Standard English stop words and a conservative set of generic research terms were removed.

The resulting corpus was represented using term frequency–inverse document frequency (TF-IDF) weighting with a minimum document frequency of 5, a maximum document-frequency threshold of 0.85, a maximum vocabulary size of 5, 000 terms, sublinear term-frequency scaling, and L2 normalization. NMF was fitted with coordinate descent and Frobenius loss using NNDSVD-based initialization. A six-component solution (K = 6) was used as a matched-resolution comparator to the primary six-topic LDA model rather than as an independently optimized estimate of the number of latent themes. Sensitivity analyses across K = 4–8 examined reconstruction error, mean normalized pointwise mutual information (NPMI) coherence of the top 10 terms, topic diversity, and matched-topic stability across random seeds 7, 42, and 2026. Because NNDSVD-based initialization yielded identical matched-topic solutions across the tested random-state settings, seed stability was 1.0 across K = 4–8 and was treated as a descriptive robustness check rather than a primary criterion for model selection. Higher-K solutions yielded modestly higher coherence but greater thematic subdivision; K = 6 was therefore retained for the principal cross-model comparison because it preserved high topic diversity and provided an interpretable matched-resolution solution. Complete sensitivity results across K = 4–8 are provided in **Supplementary Table S2**.

### 2.10 Topic prevalence, temporal trends, and diagnostics

Because LDA represents each document as a mixture of topics, overall prevalence was estimated by summing document-level topic probabilities. Probability sums were reported as equivalent-document counts and percentages of total topic probability. Annual mean topic probabilities were calculated for 2000-2026, and three-year moving averages were used to display smoothed trajectories.

Formal temporal trends were estimated with document-count-weighted linear regression, using year as the predictor and annual mean topic probability as the outcome. Slopes were converted to percentage-point change per decade. Two-sided 95% confidence intervals were calculated, and P values were adjusted across the six topics with the Benjamini-Hochberg false-discovery-rate procedure (27). Document-level diagnostics included dominant-topic probability, the margin between the two leading topics, and normalized topic entropy. Conservative thresholds (dominant probability <0.40, top-two margin <0.10, or normalized entropy >0.80) were used only to flag records for manual review and not for exclusion.

### 2.11 CLR-adjusted topic relationships

Topic probabilities are compositional because the six probabilities for each document sum to one. Zero values were replaced by a pseudocount of 1×10^-8, probabilities were renormalized, and a centered log-ratio (CLR) transformation was applied (28). Pearson correlations were then calculated between CLR-transformed topic coordinates. These coefficients were interpreted as relative associations within the six-topic composition, not as causal or biologically antagonistic effects.

## 3 Results

### 3.1 Multi-database corpus, overlap, and publication growth

The three database-specific exports contained 4, 415 records: 1, 407 from WoSCC, 1, 073 from PubMed, and 1, 935 from Scopus. Hierarchical deduplication removed 1, 482 duplicate occurrences and produced 2, 933 unique publications. Of these, 614 were indexed by all three databases; 253 were shared by exactly two databases; and 2, 066 were unique to one database. Scopus contributed the largest unique component (1, 118 records), followed by WoSCC (656) and PubMed (292) (**Table 1**).

**Table 1:** Database overlap after cross-database deduplication.

Database-origin category	Unique publications	Share of corpus (%)
All three databases	614	20.9
WoSCC and Scopus only	86	2.9
WoSCC and PubMed only	50	1.7
PubMed and Scopus only	117	4.0
WoSCC only	656	22.4
PubMed only	292	10.0
Scopus only	1118	38.1
Total	2933	100.0

The 2, 933 publications were distributed across 885 sources. Annual output increased from three publications in 2000 to 197 in 2019, 254 in 2020, and a maximum of 346 in 2021. Output remained high in 2022 (329) and 2023 (266), declined to 234 in 2024 and 195 in 2025, and reached 106 by the partial 2026 endpoint. The estimated annual growth rate was 14.7%. Bradford analysis identified 33 core sources, 141 sources in the second zone, and 711 sources in the third zone. The exploratory cumulative logistic model approached an asymptote of approximately 3, 228 publications, while the annual life-cycle model estimated a fitted peak near 2021.4 (R²=0.928). These exploratory models are consistent with rapid expansion followed by a possible transition toward maturation; however, the apparent recent decline should be interpreted cautiously because the 2026 observation is incomplete and recent publication counts may also be affected by database indexing lag (**Figure 1**).

**Figure 1:**
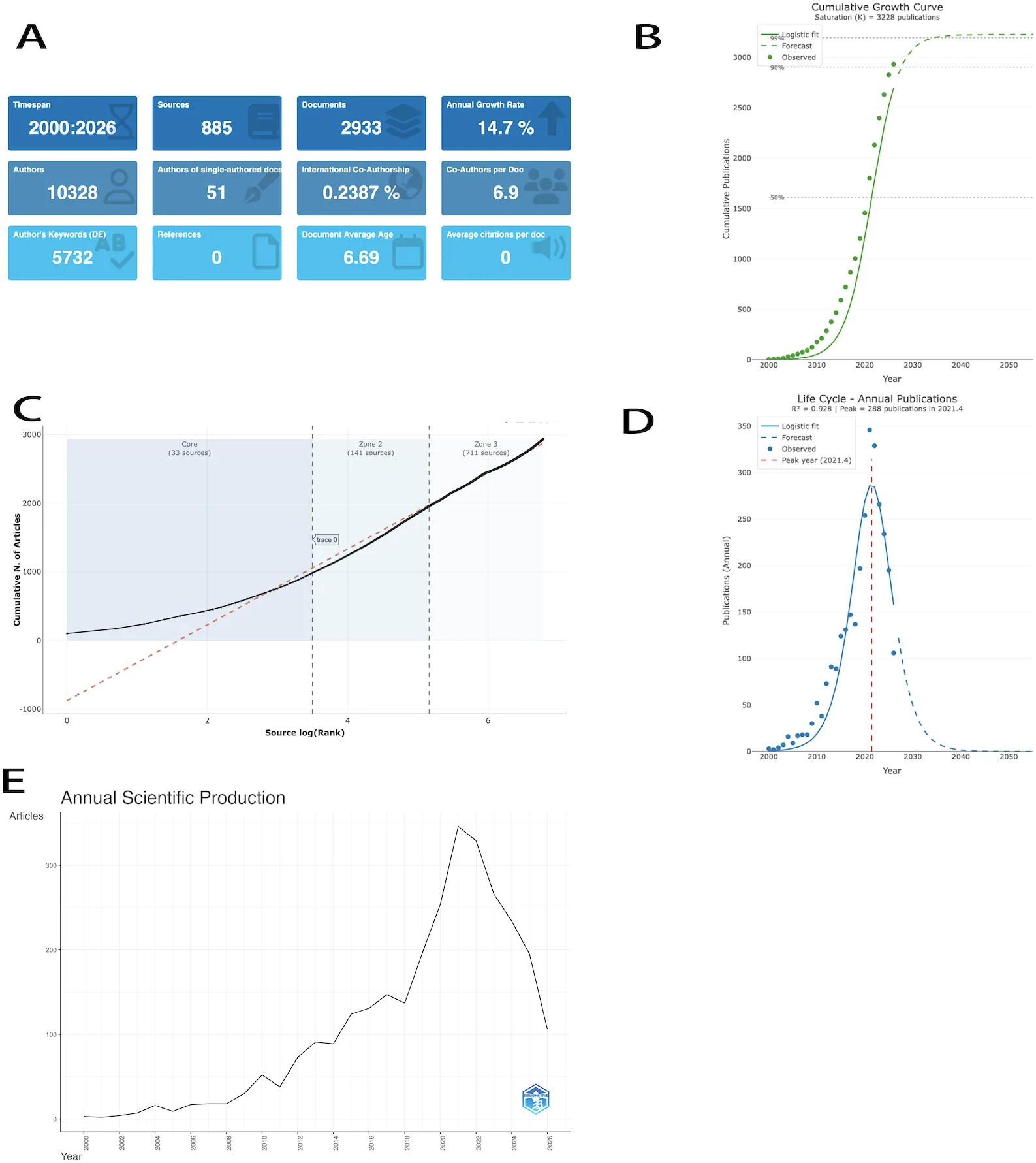
Multi-database publication characteristics and growth, 2000-2026. **(A)** Summary bibliometric indicators for the 2, 933-document corpus. **(B)** Exploratory cumulative logistic growth curve. **(C)** Bradford source zones. **(D)** Exploratory annual life-cycle model. **(E)** Observed annual scientific production. The 2026 observation is partial, and recent-year counts may also be affected by database indexing lag; therefore, the apparent late-period decline should be interpreted cautiously.

### 3.2 Country and regional collaboration

The CiteSpace country/region network contained 175 nodes and 76 links (density=0.005); the largest connected component included 47 nodes (26.9%) after minimum spanning tree pruning (**Figure 2**). The sparse backbone indicates that a limited set of international bridges connects a much larger collection of weakly integrated or isolated national nodes.

**Figure 2:**
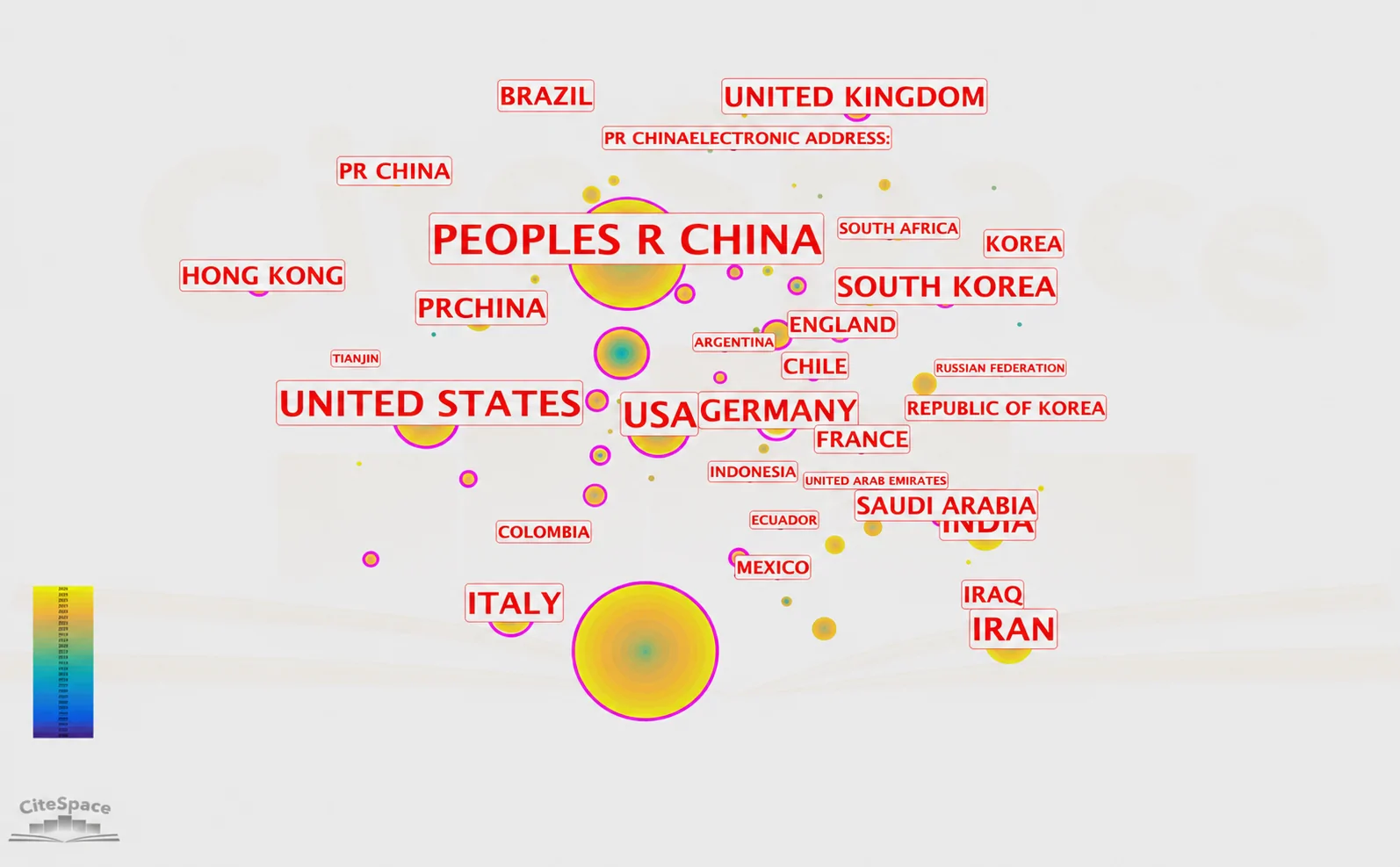
Country and regional collaboration network based on the deduplicated WoSCC-PubMed-Scopus corpus. Node size reflects publication activity, links indicate collaboration, concentric colors encode time, and purple rims indicate higher betweenness centrality. Network: N = 175, E = 76, density=0.005; largest connected component=47 nodes (26.9%); pruning=minimum spanning tree. Country aliases were consolidated in **Table 2**.

After consolidation of country aliases, China was the largest contributor with 1, 549 publications, followed by the United States (413), Japan (219), Iran (182), Italy (123), India (116), South Korea (106), Germany (100), the United Kingdom (74), and Australia (51) (**Table 2**). The United States had the highest TLS (405), compared with China (240), indicating that publication volume and international connectivity were not equivalent. The United Kingdom (TLS = 144), Iran (150), Germany (131), and Italy (126) also showed strong collaborative connectivity relative to output.

**Table 2:** Top 10 countries or regions in the harmonized multi-database collaboration network.

Rank	Country/region	Documents	TLS	Average publication year
1	China	1549	240	2020.20
2	United States	413	405	2018.14
3	Japan	219	87	2014.83
4	Iran	182	150	2021.65
5	Italy	123	126	2019.36
6	India	116	105	2021.70
7	South Korea	106	64	2015.85
8	Germany	100	131	2018.42
9	United Kingdom	74	144	2018.73
10	Australia	51	64	2019.18

### 3.3 Institutional collaboration

The institution network comprised 216 nodes and 87 links (density=0.0037), and the largest connected component contained 10 nodes (4.6%) (**Figure 3**). This was the most fragmented level of the collaboration structure, indicating that most institutional groups were connected through small regional or project-specific components rather than a cohesive global network.

**Figure 3:**
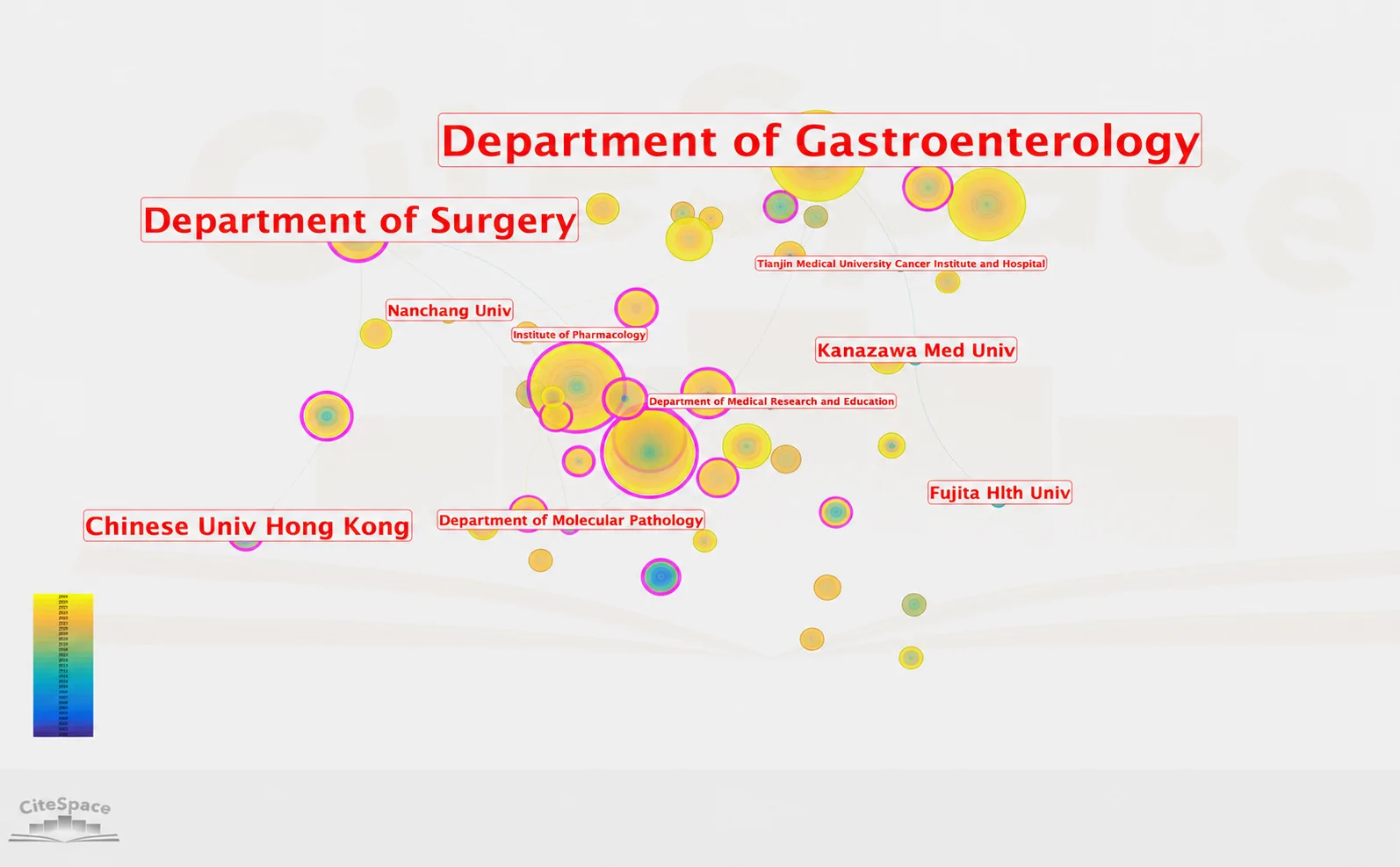
Institutional collaboration network based on the deduplicated three-database corpus. Network: N = 216, E = 87, density=0.0037; largest connected component=10 nodes (4.6%); pruning=minimum spanning tree. The software-rendered map contains some department-level nodes; **Table 3** reports harmonized organization-level entities.

After exclusion of generic department-only and incomplete organization labels, Nanjing Medical University ranked first by publication output (70 documents), followed by Central South University (55), Islamic Azad University (54), Shandong University (47), Fudan University (44), and Shanghai Jiao Tong University (43) (**Table 3**). Islamic Azad University had the highest TLS among the leading institutions (204), followed by Nanjing Medical University (113) and Shandong University (83). The National Cancer Center-Japan showed an earlier average publication year (2013.39), consistent with its established contribution to gastric mucosal methylation and H. pylori-associated epigenetic risk research.

**Table 3:** Top 10 harmonized institutions by publication output.

Rank	Institution	Documents	TLS	Average publication year
1	Nanjing Medical University	70	113	2020.27
2	Central South University	55	67	2020.07
3	Islamic Azad University	54	204	2021.46
4	Shandong University	47	83	2019.98
5	Fudan University	44	66	2020.02
6	Shanghai Jiao Tong University	43	58	2019.09
7	Jilin University	41	19	2019.59
8	China Medical University	39	36	2018.87
9	National Cancer Center - Japan	38	42	2013.39
10	Zhengzhou University	32	17	2020.19

### 3.4 Author productivity and collaboration

The author network contained 265 nodes and 95 links (density=0.0027), with 17 authors (6.4%) in the largest component (**Figure 4**). The topology indicates a field organized primarily through specialized teams, with limited bridging among established research groups.

**Figure 4:**
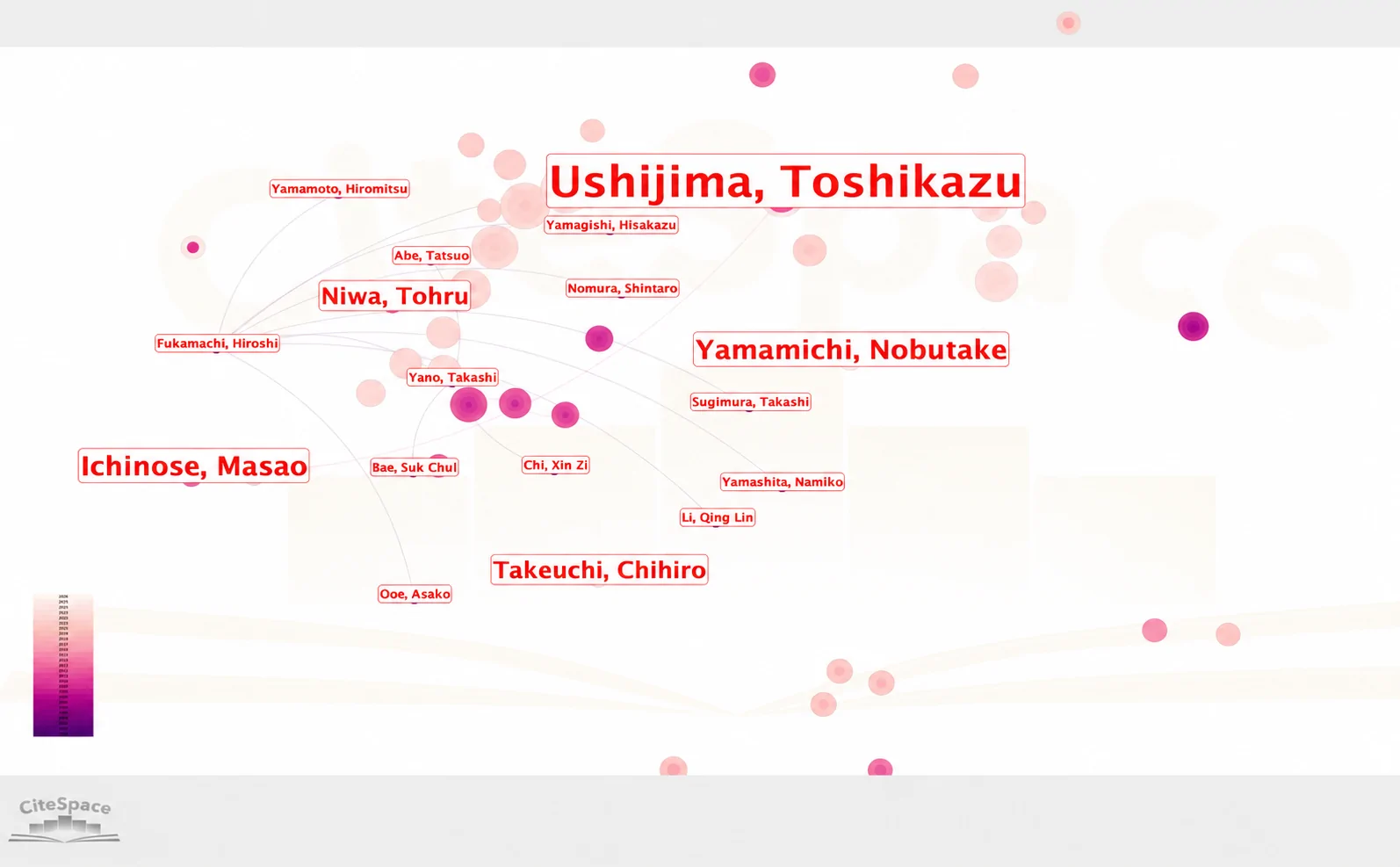
Author collaboration network based on the deduplicated WoSCC-PubMed-Scopus corpus. Network: N = 265, E = 95, density=0.0027; largest connected component=17 nodes (6.4%); pruning=minimum spanning tree. The sparse network indicates that the literature is organized through multiple specialized teams rather than one integrated author community.

Toshikazu Ushijima was the most prolific author with 25 publications, followed by Milad Ashrafizadeh and Ali Zarrabi with 20 each (**Table 4**). Zarrabi had the highest TLS (108), followed by Ashrafizadeh (100), Kiavash Hushmandi (96), Tomomitsu Tahara (87), and Tomoyuki Shibata (86). Earlier average publication years characterized authors associated with classical methylation and gastric mucosal risk research, whereas several highly connected ncRNA-focused teams had average publication years after 2021. Common names such as Yan Zhang and Jing Wang were interpreted cautiously because bibliographic disambiguation cannot fully resolve homonyms.

**Table 4:** Top 10 authors by publication output in the harmonized multi-database corpus.

Rank	Author	Documents	TLS	Average publication year
1	Ushijima, Toshikazu	25	74	2015.96
2	Ashrafizadeh, Milad	20	100	2021.20
3	Zarrabi, Ali	20	108	2021.50
4	Ghafouri-Fard, Soudeh	16	26	2022.06
5	Hushmandi, Kiavash	16	96	2021.25
6	Zhang, Yan	16	21	2019.44
7	Tahara, Tomomitsu	15	87	2012.33
8	Wang, Jing	15	2	2020.07
9	Mirzaei, Sepideh	13	79	2021.77
10	Shibata, Tomoyuki	13	86	2011.38

### 3.5 Keyword architecture and thematic hotspots

The multi-database keyword network showed a dense conceptual core linking gastric cancer with ncRNA regulation, cell proliferation, signal transduction, apoptosis, gene expression, inflammation, DNA methylation, metastasis, and the tumor microenvironment (**Figure 5A**). Standardized term counts were led by gastric cancer (2, 613 documents), microRNA (1, 301), cell proliferation (922), signal transduction (871), apoptosis (799), gene expression (711), carcinogenesis (687), protein expression (629), inflammation (605), and upregulation (587) (**Table 5**).

**Figure 5:**
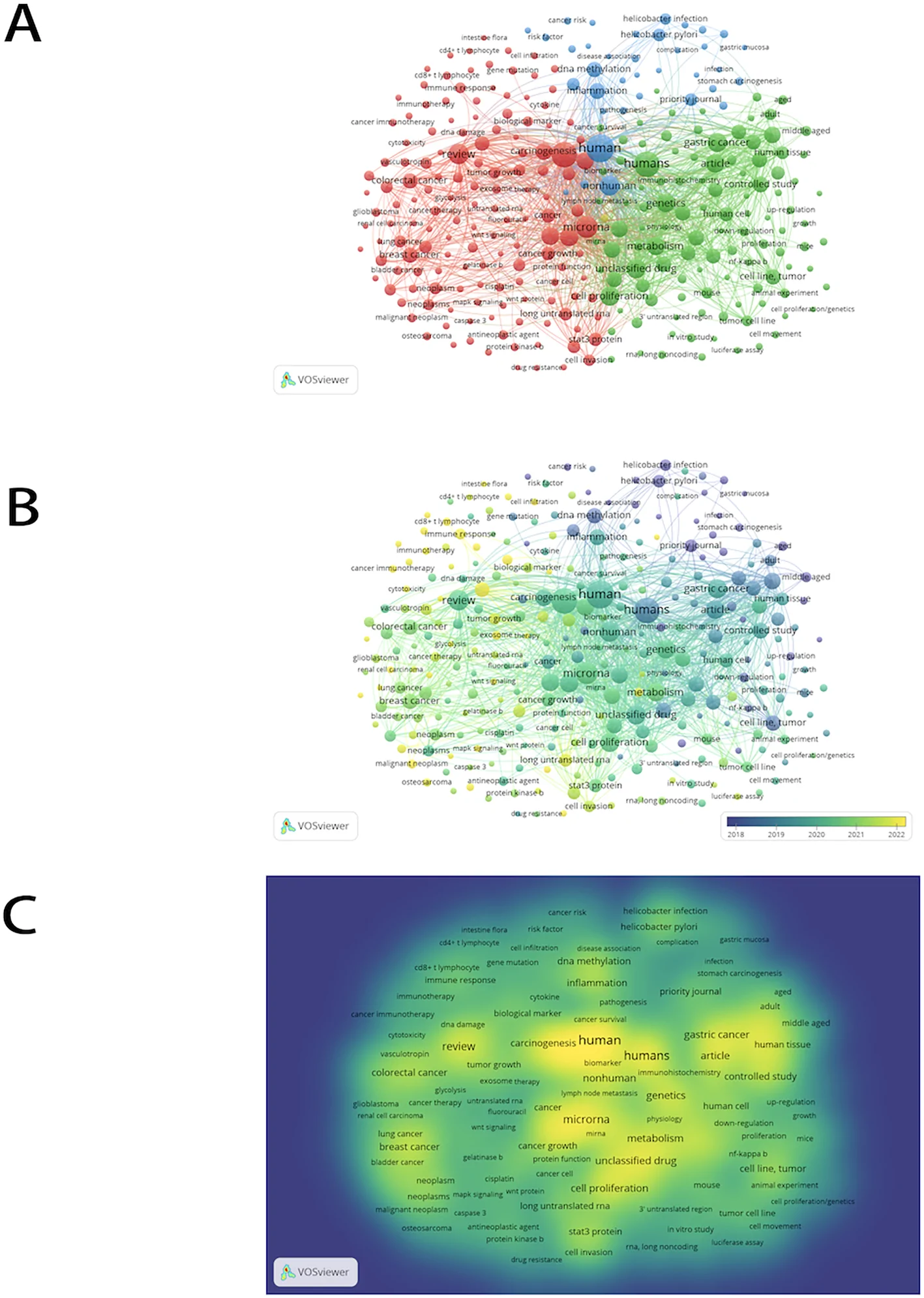
Multi-database keyword structure visualized with VOSviewer. **(A)** Network visualization of co-occurrence clusters. **(B)** Temporal overlay, with color representing average publication year. **(C)** Density visualization. The harmonized term set combined author keywords, WoSCC Keywords Plus, Scopus indexed keywords, and PubMed MeSH/other terms.

**Table 5:** Top 10 standardized terms in the multi-database keyword co-occurrence network.

Rank	Standardized term	Documents	TLS	Average publication year
1	gastric cancer	2613	68878	2019.40
2	microrna	1301	40324	2020.20
3	cell proliferation	922	33673	2020.27
4	signal transduction	871	32253	2020.00
5	apoptosis	799	28600	2019.93
6	gene expression	711	27919	2020.47
7	carcinogenesis	687	26271	2019.89
8	protein expression	629	24855	2020.00
9	inflammation	605	17078	2019.60
10	upregulation	587	25080	2020.53

The overlay view indicated that H. pylori and DNA methylation tended to have earlier average publication years, whereas tumor microenvironment, cell migration, epithelial-mesenchymal transition, lncRNA, IL-6, and resistance-related terms were concentrated in more recent years (**Figure 5B**). The density map confirmed gastric cancer, microRNA, proliferation, expression, inflammation, and signaling as the principal high-density regions (**Figure 5C**). The concordance between the multi-database keyword map and the WoSCC LDA trajectories supports a broad shift from isolated epigenetic lesions toward interacting regulatory systems.

### 3.6 Keyword bursts and changes in research emphasis

The 25 strongest keyword bursts traced an early emphasis on database indexing for gastric disease, experimental models, infection, and epigenetic regulation (**Figure 6**). The largest burst strengths were observed for priority journal (99.85; 2004-2020), humans (82.60; 2003-2014), cell line (61.35; 2007-2018), Helicobacter pylori (53.73; 2003-2015), RNA interference (51.93; 2008-2018), and DNA methylation (47.18; 2001-2013). Long-duration bursts for stomach neoplasms, H. pylori, aged, gene-expression regulation, and RNA interference are consistent with the field’s infection-methylation and experimental-model foundations.

**Figure 6:**
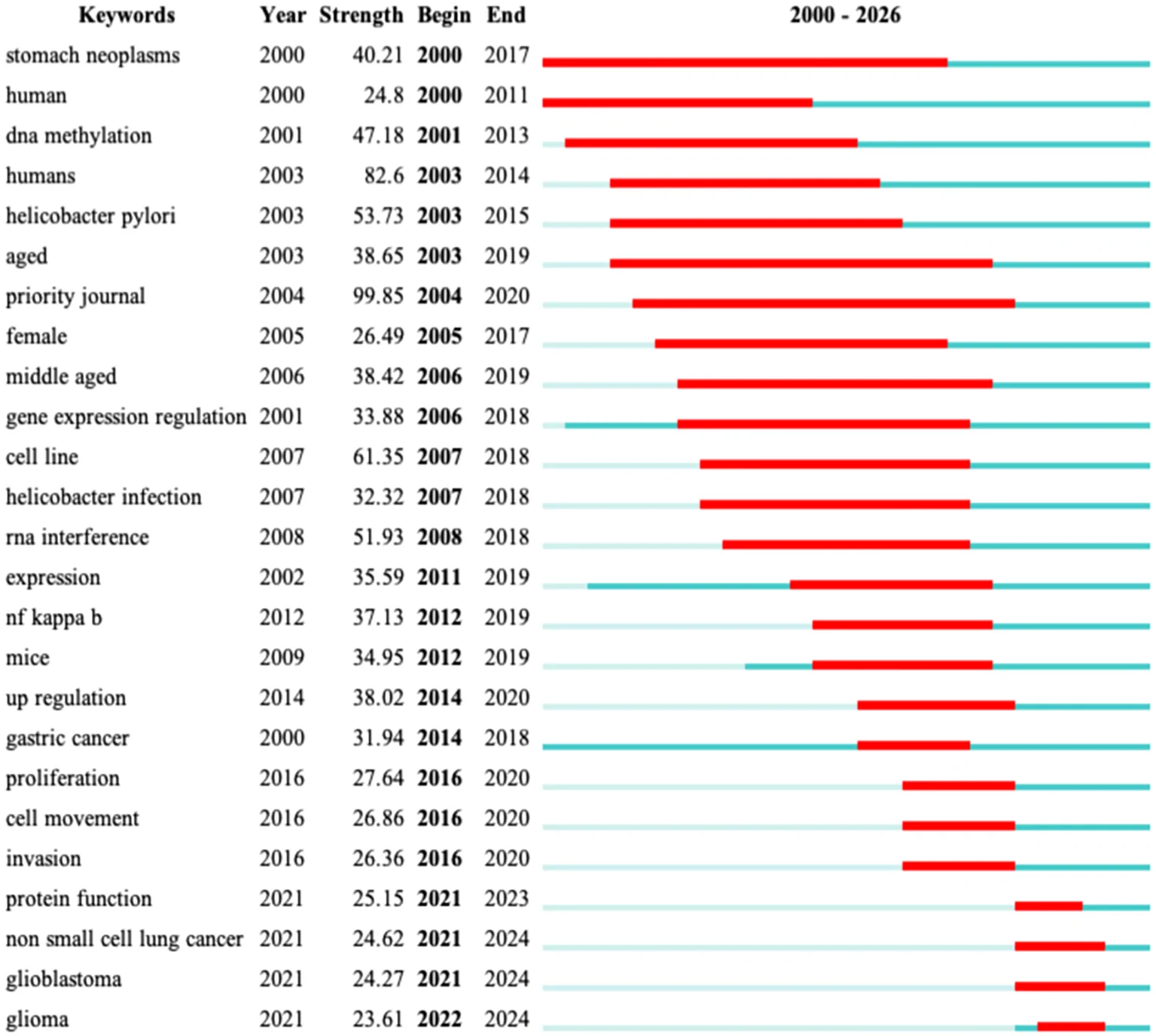
Top 25 terms with the strongest occurrence bursts, 2000-2026. Red segments indicate burst intervals. Early bursts emphasize gastric-disease indexing, H. pylori, DNA methylation, experimental models, and RNA interference. Recent cross-cancer terms should be interpreted as methodological or indexing spillover within the pooled database vocabulary.

The middle period included NF-kappa B, upregulation, proliferation, cell movement, and invasion, indicating a transition toward signaling and malignant phenotypes. The most recent bursts were protein function (2021–2023), non-small-cell lung cancer and glioblastoma (2021–2024), and glioma (2022–2024). These late cross-cancer terms likely reflect methodological transfer and broad indexing spillover rather than a gastric-cancer-specific thematic replacement. No burst in the displayed top 25 remained active through the 2026 endpoint.

### 3.7 LDA model selection and assignment characteristics

The nested LDA analysis included 808 WoSCC publications represented by 2, 315 terms. Perplexity decreased from 920.39 at K = 4 to 761.01 at K = 12, whereas coherence, minimum topic size, and stability deteriorated as K increased beyond the lower range (**Figure 7A**). At K = 6, the minimum topic size remained 93 documents, compared with 59 at K = 7 and 41 at K = 12. Random-seed stability was comparable for K = 5 and K = 6 (0.554 and 0.546) and declined at K = 7 (0.464). Together with the emergence of six nonredundant biomedical themes, these results supported K = 6 and seed 7.

**Figure 7:**
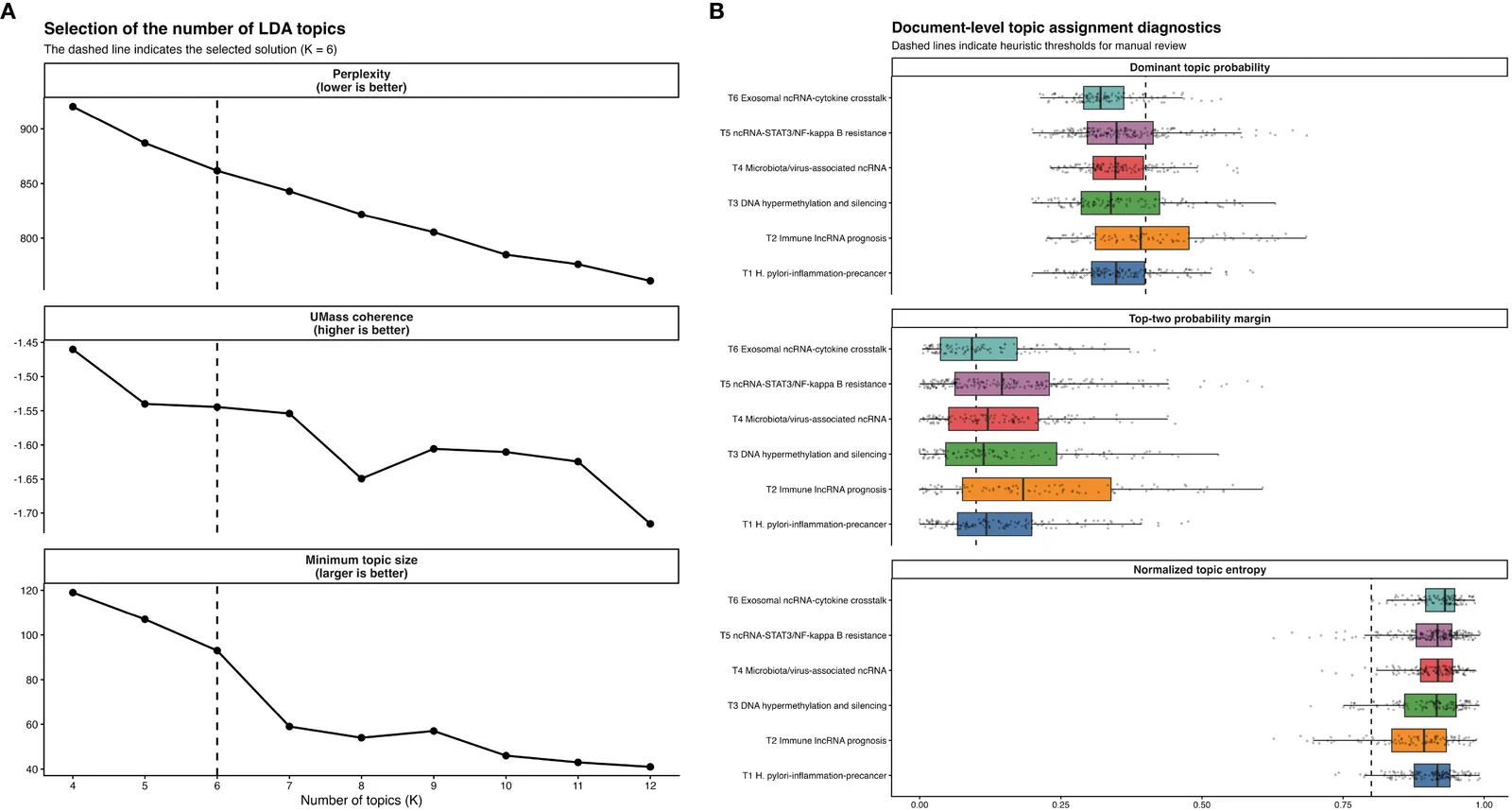
**(A)**. LDA model validation and selection of the number of topics. Candidate solutions from K = 4 to K = 12 were compared using perplexity, UMass coherence, and minimum topic size. The dashed line marks the selected K = 6 solution. **(B)**. Document-level LDA assignment diagnostics. The panels show dominant-topic probability, the top-two probability margin, and normalized topic entropy. Dashed lines are conservative manual-review thresholds and were not exclusion criteria.

Document-level diagnostics demonstrated substantial mixed-topic membership (**Figure 7B**). The overall median dominant-topic probability was 0.343, the median top-two margin was 0.123, and the median normalized entropy was 0.917. Under deliberately conservative review thresholds, 760 of 808 documents (94.06%) were flagged as mixed-topic records. This pattern was interpreted as expected thematic overlap rather than model failure and justified probability-weighted downstream analyses.

### 3.8 Six latent topics and probability-weighted prevalence

The final model produced six clinically interpretable topics (**Figure 8**). Topic 1 represented H. pylori-driven chronic inflammation and precancerous mucosal progression. Topic 2 captured immune-related lncRNA signatures and prognostic stratification. Topic 3 represented inflammation-associated DNA hypermethylation and tumor-suppressor silencing. Topic 4 combined microbiota/virus-associated ncRNA regulation with biomarkers and precision medicine. Topic 5 represented ncRNA-mediated STAT3/NF-kappa B signaling in tumor progression and drug resistance. Topic 6 represented exosomal ncRNA-cytokine crosstalk in immune remodeling, metastasis, and stemness.

**Figure 8:**
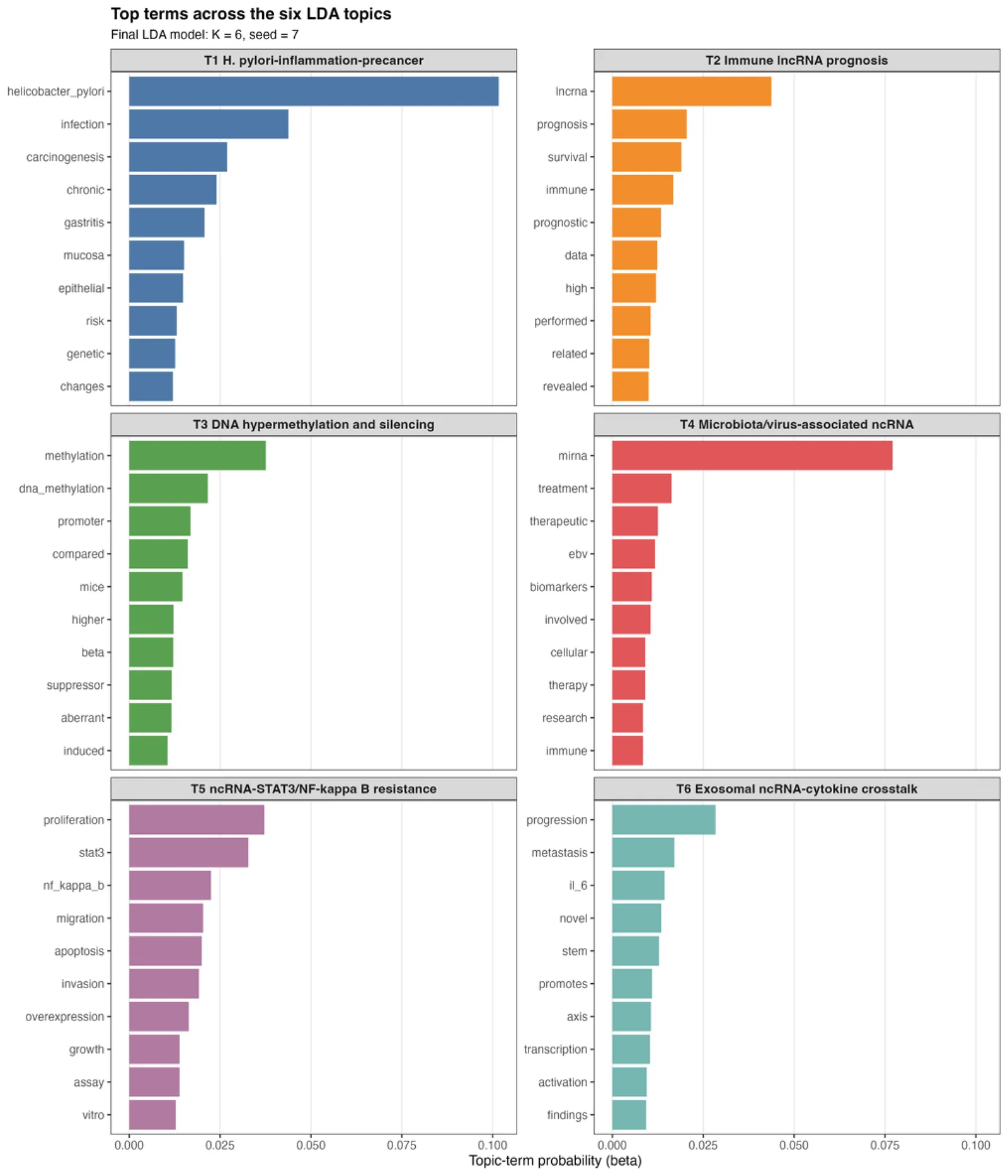
Highest-probability terms across the six topics in the final K = 6, seed=7 LDA model. Bar length represents topic-term probability. Topic labels were assigned after review of top terms and representative publications.

Probability-weighted prevalence was balanced across topics (**Figure 9** and **Table 6**). Here, “equivalent documents” refers to the sum of document-level topic probabilities for a given topic across all publications, rather than the number of publications exclusively assigned to that topic. Topic 5 was the largest at 147.5 equivalent documents (18.26%), followed by Topic 4 at 136.5 (16.90%), Topic 6 at 132.9 (16.45%), Topic 1 at 132.0 (16.34%), Topic 2 at 130.3 (16.13%), and Topic 3 at 128.7 (15.93%). The 2.33-percentage-point range indicates a diversified thematic ecosystem rather than a single dominant paradigm.

**Figure 9:**
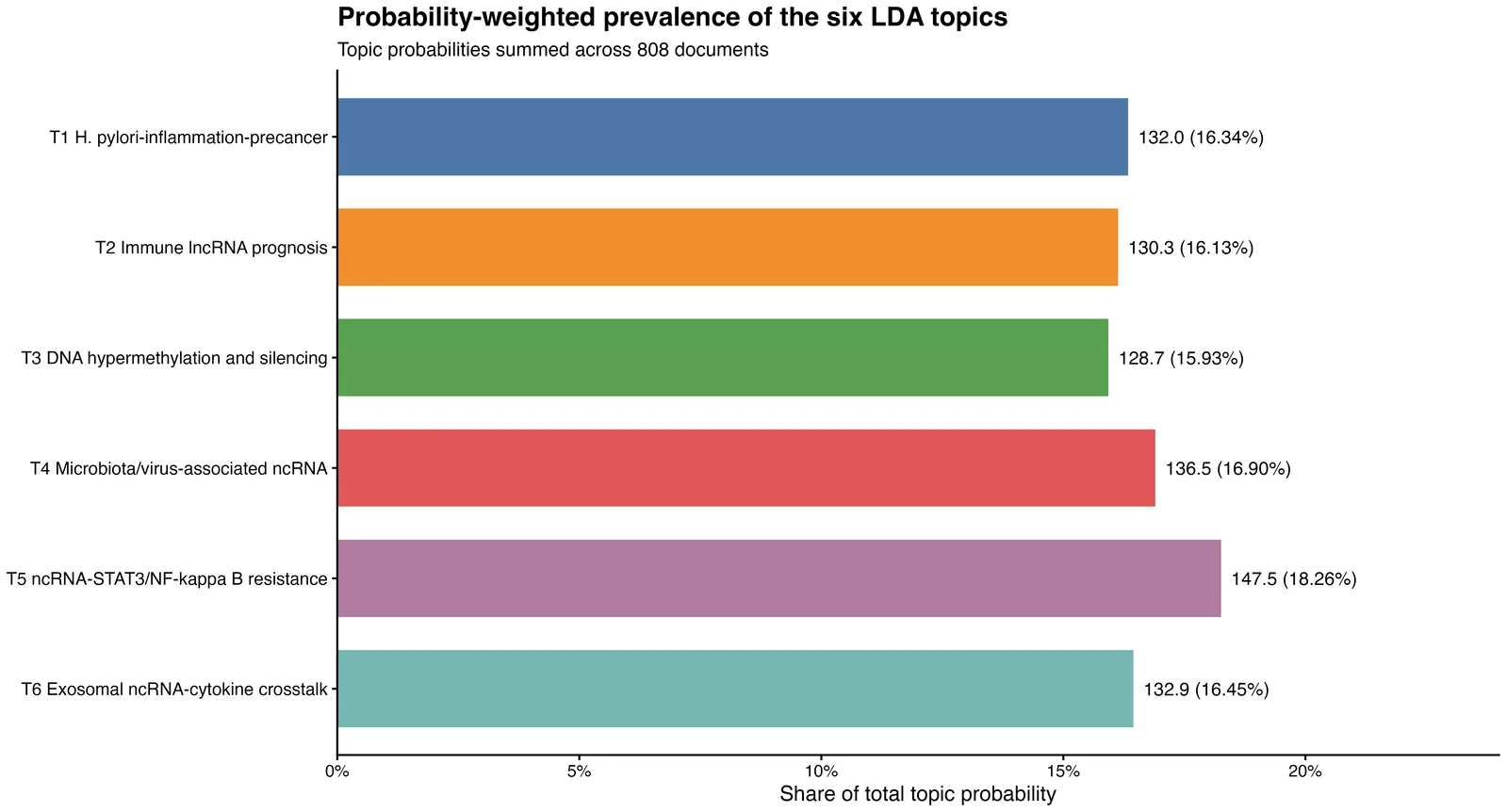
Probability-weighted prevalence of the six LDA topics. Topic probabilities were summed across all 808 WoSCC documents, preserving mixed-topic membership. Labels show equivalent-document counts and shares of total topic probability.

**Table 6:** Probability-weighted prevalence and temporal trends of the six WoSCC-based LDA topics.

Topic	Short label	Equivalent documents (share)	Change/decade, pp	95% CI	FDR-adjusted P
T1	H. pylori-inflammation-precancer	132.0 (16.34%)	-5.62	-7.62 to -3.61	1.00×10^-5
T2	Immune lncRNA prognosis	130.3 (16.13%)	+5.03	3.26 to 6.81	1.00×10^-5
T3	DNA hypermethylation-silencing	128.7 (15.93%)	-9.53	-11.37 to -7.69	1.26×10^-9
T4	Microbiota/virus-associated ncRNA	136.5 (16.90%)	+4.06	2.10 to 6.03	3.43×10^-4
T5	ncRNA-STAT3/NF-kappa B resistance	147.5 (18.26%)	+1.81	-1.34 to 4.95	0.247
T6	Exosomal ncRNA-cytokine crosstalk	132.9 (16.45%)	+4.24	3.16 to 5.33	1.08×10^-7

### 3.9 Temporal evolution of latent themes

The longitudinal thematic composition showed a marked shift in the relative distribution of research attention, from classical methylation- and infection-focused studies toward regulatory, immunological, microbial/viral, and translational themes (**Figure 10**). Topic 3 dominated the early literature but contracted substantially. Topic 1 remained prominent during the early and intermediate periods and then declined. Topics 2, 4, and 6 gained momentum after the mid-2010s, whereas Topic 5 rose through the late 2010s and subsequently showed a partial decline.

**Figure 10:**
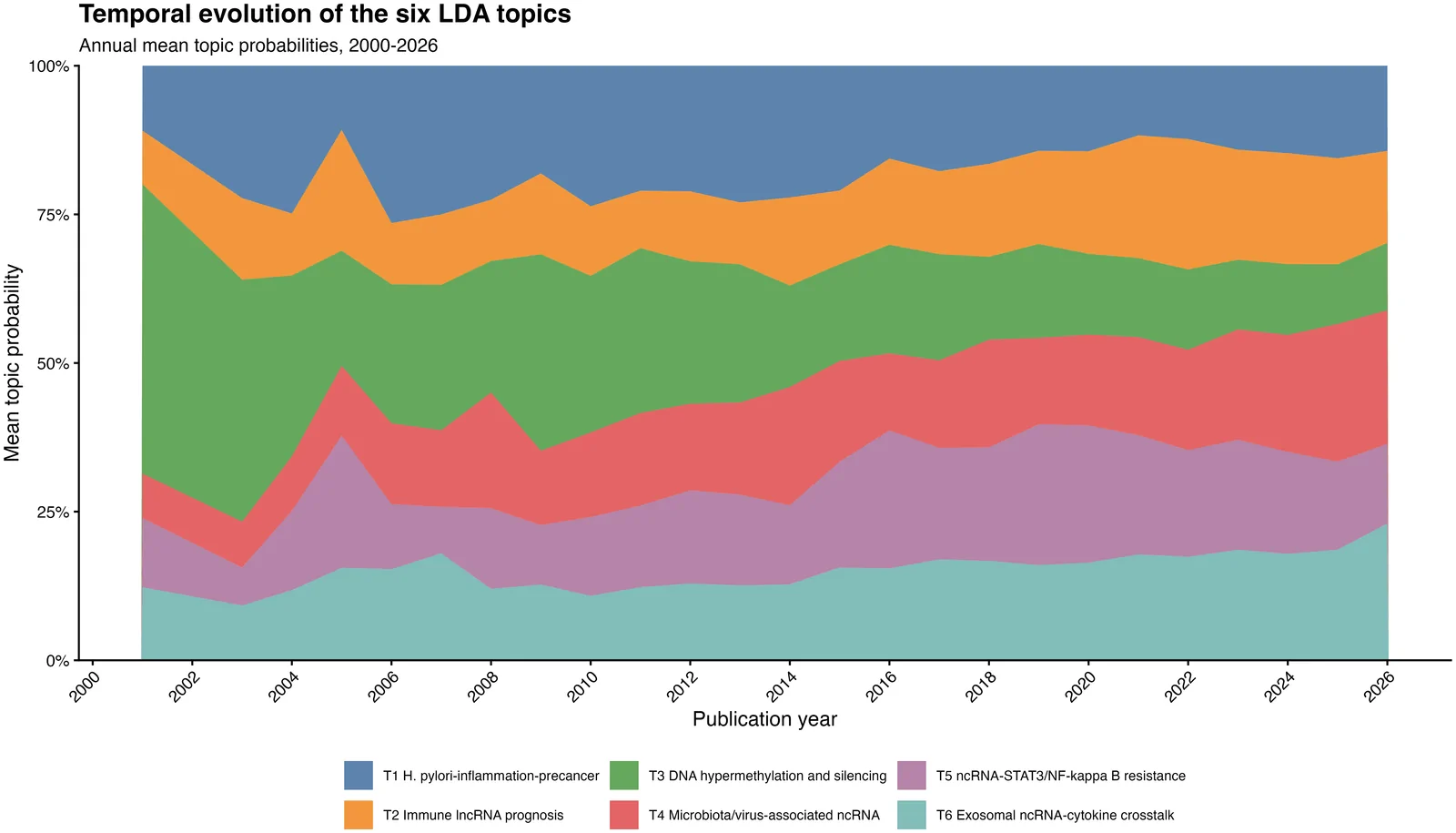
Temporal evolution of the six LDA topics. The stacked area chart shows annual mean topic probabilities from 2000 to 2026; each year sums to 100%. Early-year fluctuations reflect small annual denominators.

Topic-specific moving averages clarified the individual trajectories (**Figure 11**). Topic 3 fell from approximately 45% in the earliest years to about 11% by 2026. Topic 1 peaked near one quarter of topic probability in the mid-2000s and then declined. Topic 2 increased most strongly after the mid-2010s and plateaued near 2020–2021 before moderating. Topic 4 increased over the long term and exceeded 20% by 2026, while Topic 6 accelerated in recent years. Topic 5 was nonmonotonic, peaking around 2019–2020 before receding.

**Figure 11:**
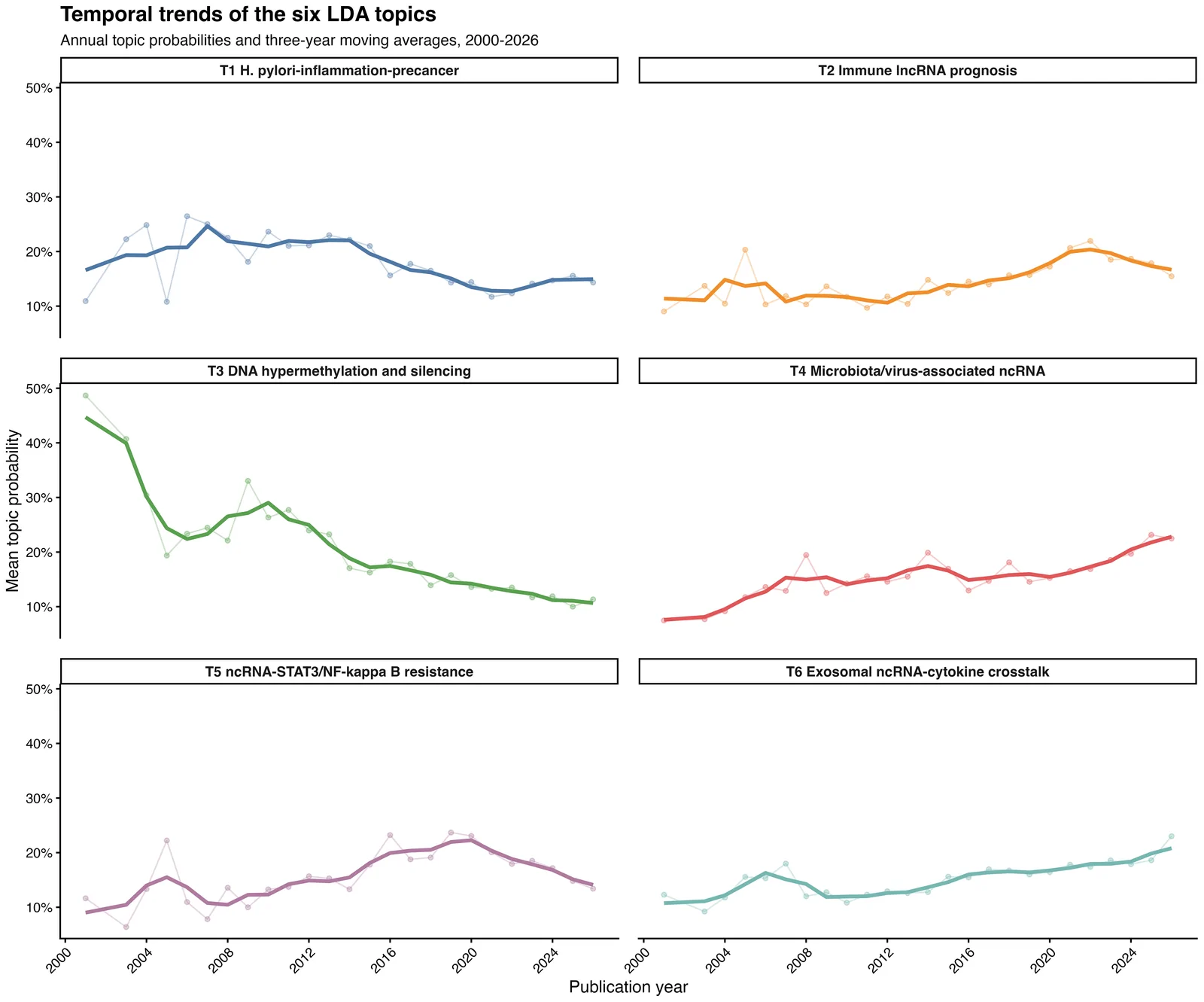
Topic-specific temporal trends. Light points and lines show annual mean topic probabilities; thick lines show three-year moving averages. The plots show decline in infection-methylation themes, growth of immune, microbial/viral, and exosomal themes, and a nonmonotonic resistance-signaling trajectory.

### 3.10 Statistical trends

Document-count-weighted regression confirmed significant decreases in Topic 1 and Topic 3 (**Figure 12**). Topic 1 declined by 5.62 percentage points per decade (95% CI, -7.62 to -3.61; FDR-adjusted P = 1.00×10^-5), whereas Topic 3 declined by 9.53 points per decade (95% CI, -11.37 to -7.69; FDR-adjusted P = 1.26×10^-9).

**Figure 12:**
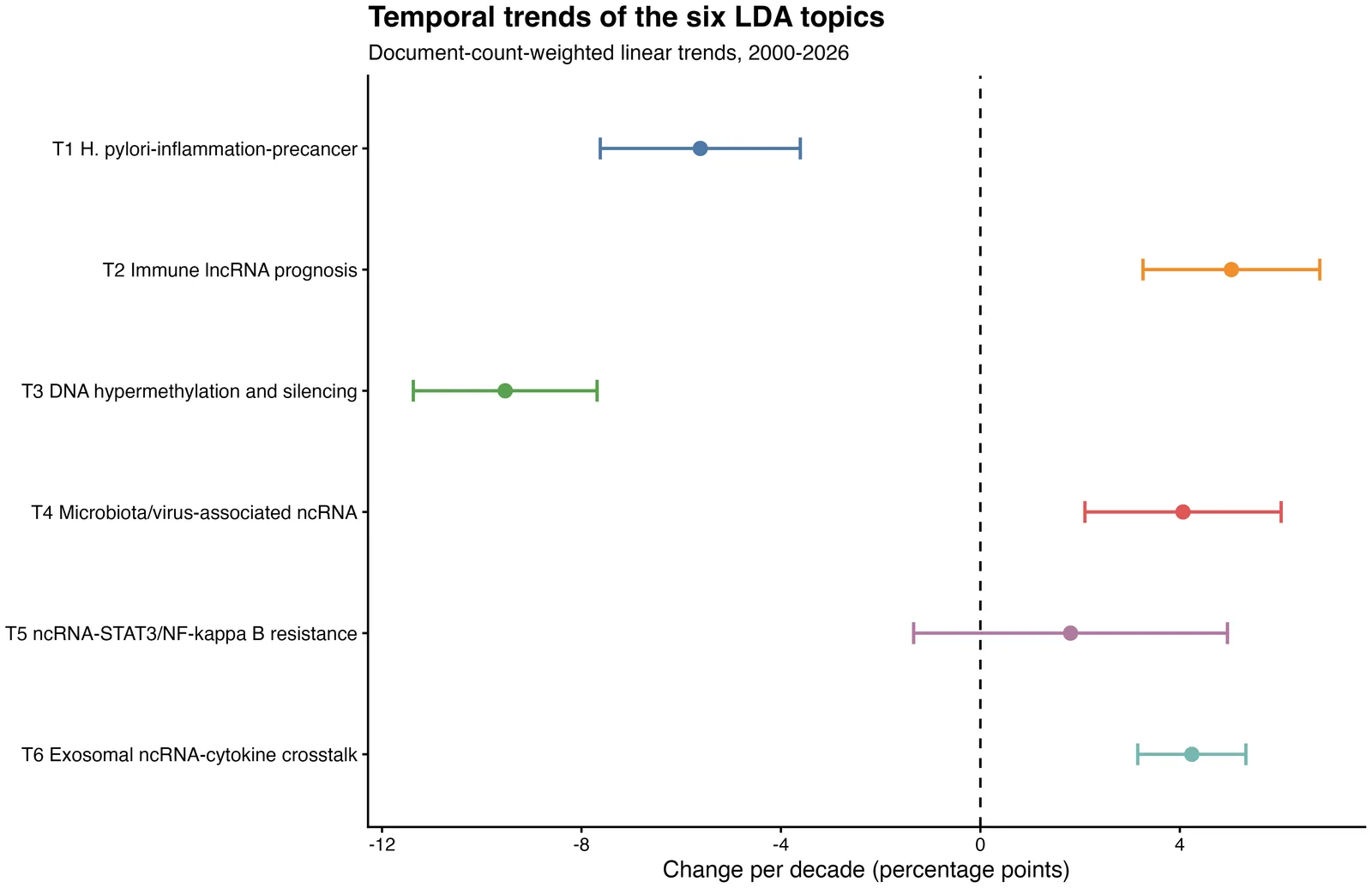
Document-count-weighted linear trends in annual topic probability. Points represent percentage-point change per decade and horizontal bars represent 95% confidence intervals. The dashed line marks no change.

Three topics increased significantly: Topic 2 by 5.03 percentage points per decade (95% CI, 3.26-6.81; FDR-adjusted P = 1.00×10^-5), Topic 4 by 4.06 points (95% CI, 2.10-6.03; FDR-adjusted P = 3.43×10^-4), and Topic 6 by 4.24 points (95% CI, 3.16-5.33; FDR-adjusted P = 1.08×10^-7). Topic 5 increased by 1.81 points per decade, but the confidence interval crossed zero (95% CI, -1.34 to 4.95; FDR-adjusted P = 0.247), confirming that its rise-and-fall trajectory was not well summarized by a single linear slope.

### 3.11 Relative relationships among topics

CLR-adjusted analysis revealed a limited number of positive relative relationships and several negative compositional associations (**Figure 13**). The strongest positive coefficient was between Topic 5 and Topic 6 (r=0.22, P<0.001), linking intracellular ncRNA-STAT3/NF-kappa B resistance mechanisms with exosomal ncRNA-cytokine and metastatic processes. Topic 1 showed weak positive associations with Topic 3 and Topic 4 (both r=0.11, P<0.01), consistent with links among infection-driven mucosal injury, methylation, and microbial or viral regulation.

**Figure 13:**
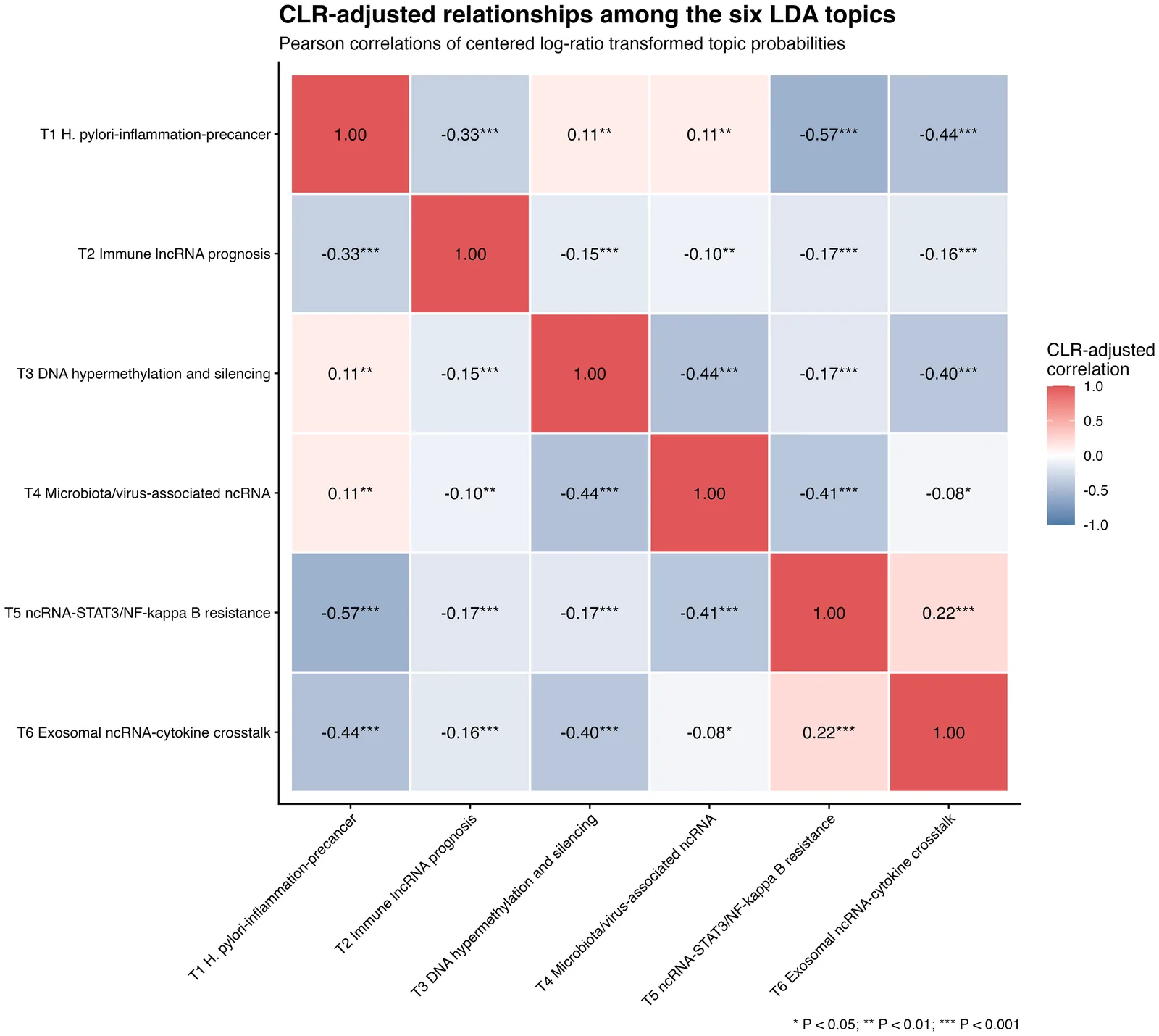
CLR-adjusted relationships among the six LDA topics. Values are Pearson correlations of centered log-ratio-transformed document-level probabilities. Positive and negative coefficients represent relative compositional associations and should not be interpreted as causal molecular effects. P<0.05; P<0.01; P<0.001.

The strongest negative relative association was between Topic 1 and Topic 5 (r=-0.57, P<0.001), followed by Topic 1 and Topic 6 (r=-0.44), Topic 3 and Topic 4 (r=-0.44), Topic 4 and Topic 5 (r=-0.41), and Topic 3 and Topic 6 (r=-0.40). These values represent relative allocation within a fixed six-topic composition and do not indicate biological antagonism or mutual exclusivity.

### 3.12 Independent multi-database validation using NMF

To assess whether the thematic structure identified by the WoSCC-based LDA model was reproducible across the broader integrated corpus and under a different modeling framework, non-negative matrix factorization (NMF) was applied to the common title-and-abstract text of all 2, 933 unique publications. A matched-resolution six-component solution was used for direct thematic comparison with the primary six-topic LDA model. The NMF solution identified six interpretable components characterized by (1): ncRNA-related signaling and therapeutic research (2); proliferation, migration, invasion, and STAT3-associated signaling (3); *H. pylori* infection, inflammation, and gastric carcinogenesis (4); prognostic lncRNA and immune-related profiling (5); exosome-, immune-, and tumor-microenvironment interactions; and (6) DNA methylation, promoter hypermethylation, CpG-related regulation, and EBV-associated epigenetic processes (**Figure 14**). These components showed broad thematic concordance with the major biological domains recovered by the WoSCC LDA model, particularly the *H. pylori*–inflammation axis, ncRNA-associated signaling, immune/exosomal microenvironmental regulation, and DNA methylation-related epigenetic mechanisms. The correspondence was not strictly one-to-one, indicating that the independent NMF model reproduced the major thematic domains while partitioning some cross-cutting concepts differently. Detailed thematic correspondence between the six LDA topics and the six NMF components is summarized in **Supplementary Table S3**.

**Figure 14:**
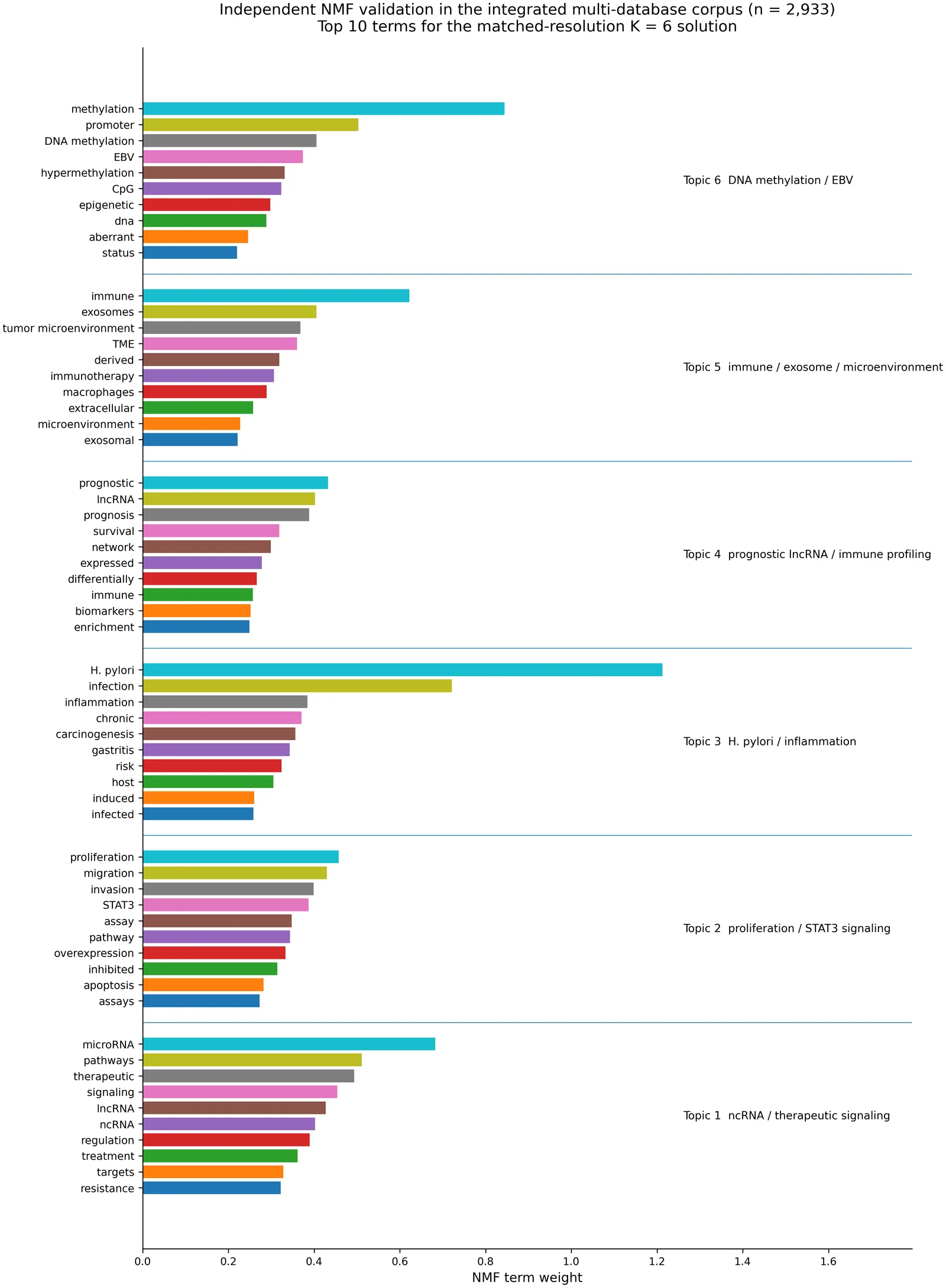
Independent validation of thematic structure using non-negative matrix factorization (NMF) in the integrated multi-database corpus. NMF was applied to the common text fields (title and abstract) of 2, 933 unique publications retrieved from Web of Science Core Collection, PubMed, and Scopus. A matched-resolution six-component solution (K = 6) was used to facilitate comparison with the primary six-topic LDA model. The figure shows the 10 highest-weight terms for each NMF component. The six components broadly captured ncRNA-related therapeutic signaling, STAT3-associated proliferation and invasion, H. pylori–associated inflammation, prognostic lncRNA and immune profiling, exosome–immune–tumor microenvironment interactions, and DNA methylation/EBV-related epigenetic processes. NMF was used as an independent robustness analysis rather than as a replacement for the primary LDA model.

## 4 Discussion

### 4.1 Principal findings

. This multi-database study extends the characterization of inflammation-associated epigenetic research in gastric cancer beyond a single-database perspective. The principal value of this design lies not only in expanding literature coverage, but also in showing that database choice can materially influence the observable bibliometric structure of the field, while major thematic domains remain broadly recognizable across independent analytical approaches. These findings support a distinction between database-dependent bibliometric patterns and more persistent thematic structures, while observed temporal changes are more appropriately interpreted as shifts in research emphasis rather than evidence of confirmed biological evolution.

The six-topic LDA model, although restricted to the 808-document WoSCC subset, showed broad thematic convergence with two independent analyses conducted on the full 2, 933-publication corpus. The multi-database keyword maps and the independent NMF model both recovered major domains involving *H. pylori* and inflammation, DNA methylation, ncRNA regulation, STAT3/NF-κB-associated signaling, immune and tumor-microenvironment processes, and extracellular-vesicle-related regulation. Importantly, the NMF analysis used only title and abstract fields shared across all three databases and a modeling framework mathematically distinct from LDA. This convergence indicates that the principal thematic domains identified by the nested WoSCC LDA were not confined to WoSCC-specific indexing fields or to the LDA algorithm itself. However, the correspondence was not strictly one-to-one, and the analysis does not establish that the 808-document WoSCC subset is statistically representative of all 2, 933 publications. Rather, it supports the more limited conclusion that the major thematic structure inferred from the WoSCC subset remained visible after expansion to the integrated multi-database corpus.

### 4.2 Added value of multi-database integration

The overlap structure demonstrates why database choice materially affects bibliometric conclusions. Only 614 publications were indexed by all three sources, while Scopus contributed 1, 118 unique records, WoSCC 656, and PubMed 292. PubMed added biomedical indexing through MeSH and coverage of medically oriented sources, Scopus expanded journal coverage and author-affiliation metadata, and WoSCC supplied established science-mapping fields. These complementary contributions support the use of multiple databases when the objective is to characterize a biomedical research field rather than a platform-specific literature subset (22, 23).

Multi-database integration also introduces methodological responsibilities. DOI and PMID identifiers are incomplete, title strings vary across platforms, online-first and final publication years may differ, and author and institution names can be fragmented or transliterated inconsistently. The present hierarchical deduplication and field-level provenance retained complementary metadata while preventing simple addition of overlapping records. Citation counts were deliberately not pooled, because apparent precision would obscure platform-specific coverage differences.

### 4.3 Global production and collaboration

China’s dominance in publication volume is consistent with its substantial gastric-cancer burden, investment in molecular oncology, and rapid expansion of ncRNA research (29). The United States produced fewer papers but showed the highest TLS, suggesting a stronger brokerage role across national boundaries. Japan retained a distinctive contribution through long-standing work on H. pylori-associated epigenetic field defects, methylation-based risk prediction, and gastric mucosal carcinogenesis (30, 31). Iran, Italy, India, Germany, South Korea, and the United Kingdom formed additional productive and internationally connected communities.

The network topology became progressively more fragmented from countries to institutions and authors. Only 26.9% of country nodes, 4.6% of institution nodes, and 6.4% of author nodes belonged to the largest pruned components. However, network density and component size are partly dependent on corpus size, node-selection thresholds, and minimum-spanning-tree pruning; therefore, the observed sparsity should not be interpreted solely as a direct measure of researchers’ collaborative behavior. The very low institution and author densities suggest that international visibility can coexist with limited integration among laboratories and clinical centers. This fragmentation may contribute to repeated single-center biomarker studies, variable preprocessing pipelines, and insufficient external validation. Prospective multicenter consortia should prioritize shared biospecimen standards, harmonized epigenomic workflows, cross-population replication, and clinically meaningful endpoints rather than collaboration volume alone.

### 4.4 Shifting research emphasis from infection-associated methylation to regulatory ecosystems

Early burst terms and the declining LDA trajectories of Topics 1 and 3 identify H. pylori-related inflammation, promoter hypermethylation, and tumor-suppressor silencing as the foundational paradigm of the field. These mechanisms established a continuum from chronic microbial injury to persistent epigenetic remodeling, mucosal field defects, intestinal metaplasia, and malignant transformation. Their declining relative share should not be interpreted as reduced biological importance. Instead, methylation and infection have become mature explanatory frameworks that are increasingly embedded in multi-omic classification, liquid-biopsy development, risk stratification, and systems-level models.

The recent multi-database overlay and the significant growth of Topics 2, 4, and 6 indicate a shift in research emphasis from studies centered on isolated epigenetic lesions toward studies of interacting regulatory ecosystems. ncRNAs now connect transcriptional regulation with cytokine signaling, immune-cell states, epithelial plasticity, extracellular-vesicle communication, and drug response. The positive CLR association between Topics 5 and 6 is biologically plausible because intracellular STAT3/NF-kappa B signaling, IL-6 family cytokines, exosomal cargo, stemness, metastasis, and resistance frequently co-occur within integrated mechanistic studies. Nevertheless, the association is documentary and compositional rather than causal.

### 4.5 Interpretation of keyword bursts and cross-cancer spillover

The pooled keyword-burst analysis differed from the original WoSCC-only burst profile because PubMed and Scopus contribute extensive controlled and indexed vocabulary. Generic terms such as humans, priority journal, cell line, and aged therefore achieved high burst strengths. The recent appearance of non-small-cell lung cancer, glioblastoma, and glioma should not be interpreted as replacement of gastric-cancer research. Rather, these terms indicate transfer of ncRNA, signaling, and functional-genomics methods across cancer types and illustrate the indexing spillover inherent in broad bibliographic corpora. For this reason, burst results were interpreted together with the harmonized VOSviewer map and the gastric-focused LDA subset.

### 4.6 Methodological contribution

The principal methodological contribution of this study is the separation of database coverage, topic-model consistency, and cross-model robustness within a layered analytical framework. The primary science-mapping corpus maximized literature coverage through integration of WoSCC, PubMed, and Scopus, whereas the nested WoSCC LDA corpus preserved an internally consistent combination of title, abstract, author-keyword, and Keywords Plus fields for high-resolution topic modeling. Model selection for LDA was not based on a single numerical criterion but combined perplexity, coherence, topic-size balance, random-seed stability, representative-document review, and biomedical interpretability. Probability-weighted prevalence preserved mixed-topic membership, document-count weighting reduced the influence of low-output early years, and CLR transformation addressed the compositional constraint of document-level topic probabilities.

Independent NMF analysis of the full 2, 933-publication corpus provided a third analytical layer for evaluating whether the principal LDA-derived themes persisted beyond the WoSCC subset and under a mathematically distinct topic-modeling framework. By restricting NMF input to title and abstract fields shared across all three databases, this validation reduced dependence on database-specific indexing vocabularies. The broad convergence of NMF, multi-database keyword mapping, and WoSCC-based LDA strengthens confidence that the major thematic domains were not artifacts of either a single database or a single topic-modeling algorithm. Nevertheless, this convergence should be interpreted as thematic robustness rather than proof of statistical representativeness: the LDA prevalence estimates and temporal trajectories remain specific to the screened WoSCC corpus and should not be extrapolated as exact estimates for PubMed- or Scopus-only publications.

### 4.7 Research priorities

The integrated findings suggest five priority directions for future research. First, mechanistic studies should connect *H. pylori*- and inflammation-induced epigenetic alterations with contemporary immune and stromal models, rather than treating classical methylation pathways as isolated historical mechanisms. Second, ncRNA- and extracellular-vesicle-based biomarkers should undergo prospective, multicenter, cross-platform validation with predefined endpoints and independent external cohorts. Third, biomarker studies should clearly distinguish prognostic from predictive utility and determine whether epigenetic signatures provide incremental value beyond established clinicopathological variables (32, 33). Fourth, treatment-resistance studies should incorporate serial sampling, clonal evolution, immune context, and pharmacodynamic endpoints to link epigenetic regulation with therapeutic response over time. Fifth, computational and multi-omic studies should prioritize reproducible preprocessing, harmonized nomenclature, transparent model reporting, external validation, and biologically interpretable features. Collectively, these priorities emphasize a shift from descriptive molecular associations toward externally validated, clinically actionable, and mechanistically integrated research.

### 4.8 Limitations

This study has several limitations. First, although WoSCC, PubMed, and Scopus provide complementary coverage of biomedical and multidisciplinary literature, the study did not include other bibliographic sources such as Embase, Dimensions, Crossref, or Google Scholar. Publications uniquely indexed in these sources may therefore have been missed. The three-database design was selected to balance breadth of coverage with the availability of structured bibliographic metadata required for reproducible deduplication, field harmonization, science mapping, and text analysis. In addition, the English-language restriction may underrepresent regional and non-English research. Second, database metadata differed in field completeness and indexing conventions. Author-name disambiguation, country extraction, and institution harmonization cannot be perfect, particularly for common surnames, transliterated names, affiliated hospitals, departmental units, and reorganized institutions. Third, the pooled keyword set combined author-supplied and database-generated terms; consequently, generic demographic and document-type terms and cross-cancer indexing spillovers appeared in the network and burst analysis.

Fourth, citation counts from WoSCC and Scopus were not pooled; therefore, the multi-database results emphasize productivity and connectivity rather than a unified citation-impact ranking. Fifth, the primary LDA analysis remained restricted to the screened WoSCC subset and may therefore underrepresent themes concentrated in PubMed- or Scopus-unique records. Although the independent NMF analysis of all 2, 933 publications reproduced the major thematic domains, this cross-model convergence does not establish statistical representativeness of the WoSCC subset, and the LDA-derived prevalence estimates and temporal trajectories should be interpreted as specific to that nested corpus. Sixth, the broad search required simultaneous gastric-cancer, inflammation, and epigenetic concepts, so the conclusions apply specifically to inflammation-associated gastric cancer epigenetics rather than the entirety of gastric cancer epigenetics. Seventh, 2026 represented a partial observation window, and database indexing lag may delay the appearance of recently published records. Accordingly, the 2026 publication count and any apparent late-period decline should not be interpreted as completed-year trends. Finally, LDA is a bag-of-words model, topic labels remain expert interpretations, exploratory growth curves are not forecasts, and CLR correlations do not demonstrate molecular causality.

## 5 Conclusions

Integration of WoSCC, PubMed, and Scopus produced a 2, 933-publication corpus and showed that single-database analysis would omit a substantial proportion of the observable literature. Research attention in the field has shifted from *H. pylori*-associated inflammation, promoter hypermethylation, and tumor-suppressor silencing toward a more diversified set of themes involving microRNAs, lncRNAs, microbial and viral interactions, STAT3/NF-κB signaling, extracellular-vesicle communication, tumor immunity, and treatment resistance. China led publication volume, whereas the United States showed the strongest international connectivity; institution- and author-level collaboration remained highly fragmented. The nested six-topic LDA model quantified divergent long-term thematic trajectories within the screened WoSCC corpus, while independent NMF analysis of the full 2, 933-publication corpus recovered broadly concordant thematic domains. This cross-model convergence supports the robustness of the principal thematic structure across broader database coverage and a distinct topic-modeling framework, although it should not be interpreted as proof that the WoSCC subset is statistically representative of the entire corpus. Together, the multi-database science maps, nested LDA analysis, and independent NMF validation provide a reproducible framework for characterizing mature foundations, identifying active translational interfaces, and prioritizing multicenter validation in inflammation-associated gastric cancer epigenetics.

## Data Availability

The original contributions presented in the study are included in the article/**Supplementary Material**. Further inquiries can be directed to the corresponding author.

## Supplementary material

Supplementary Table 1 Harmonized database search framework.
