# Supplementary material for: Global knowledge structure and thematic evolution in inflammation-associated gastric cancer epigenetics: a multidatabase science-mapping study with nested latent Dirichlet allocation and independent NMF validation
Source: Front Immunol. 2026 Sep 25;17:1946189. doi: 10.3389/fimmu.2026.1946189 (PMC13663662; doi:10.3389/fimmu.2026.1946189)
Supplement: Supplementary file 1 [file DataSheet5.zip › Supplementary_Materials_Final_Safe_Package/Supplementary_Tables/Supplementary_Tables_S1-S3_NOTE.docx]

# Supplementary Tables S1-S3

This folder should contain the final Supplementary Materials S1-S3 document/tables submitted with the manuscript.
