# Supplementary material for: Global knowledge structure and thematic evolution in inflammation-associated gastric cancer epigenetics: a multidatabase science-mapping study with nested latent Dirichlet allocation and independent NMF validation
Source: Front Immunol. 2026 Sep 25;17:1946189. doi: 10.3389/fimmu.2026.1946189 (PMC13663662; doi:10.3389/fimmu.2026.1946189)
Supplement: Supplementary file 1 [file DataSheet5.zip › Supplementary_Materials_Final_Safe_Package/README/README.docx]

# README - Supplementary Materials Package

This package provides supplementary materials supporting the analytical workflow described in the manuscript.

Included materials are limited to documented supplementary analyses, methodological parameter summaries, and data-processing/harmonization information described in the manuscript.

No original historical analysis scripts are provided because archived working scripts from the original analysis environment were not retained.

Raw WoSCC and Scopus bibliographic records are not redistributed because their reuse and redistribution are subject to database-provider licensing restrictions.
